# Amoebae as Targets for Toxins or Effectors Secreted by Mammalian Pathogens

**DOI:** 10.3390/toxins13080526

**Published:** 2021-07-28

**Authors:** Ascel Samba-Louaka

**Affiliations:** UMR CNRS 7267 Ecologie et Biologie des Interactions, Université de Poitiers, Pôle Biologie-Santé, Bât. B36/B37, 1 rue Georges Bonnet, TSA 51106, CEDEX 9, 86073 Poitiers, France; ascel.samba@univ-poitiers.fr

**Keywords:** amoeba, effectors, pathogens, toxins

## Abstract

Numerous microorganisms, pathogenic for mammals, come from the environment where they encounter predators such as free-living amoebae (FLA). The selective pressure due to this interaction could have generated virulence traits that are deleterious for amoebae and represents a weapon against mammals. Toxins are one of these powerful tools that are essential for bacteria or fungi to survive. Which amoebae are used as a model to study the effects of toxins? What amoeba functions have been reported to be disrupted by toxins and bacterial secreted factors? Do bacteria and fungi effectors affect eukaryotic cells similarly? Here, we review some studies allowing to answer these questions, highlighting the necessity to extend investigations of microbial pathogenicity, from mammals to the environmental reservoir that are amoebae.

## 1. Introduction

The pathogenicity of several microorganisms can be directly linked to the toxins they produce. For example, most people know of historical diseases such as Cholera and Tetanus for which the role of toxins is essential. These diseases caused, respectively, by *Vibrio cholerae* and *Clostridium tetani* result from the action of virulence factors such as the cholera toxin [1] and tetanus neurotoxins [2].

Toxins are toxic molecules produced and released by microorganisms to target other organisms. They are also powerful tools used by bacteria and fungi to promote infection. Depending on the mode of action of toxins or their targets, microorganisms expressing these molecules can damage their hosts. Effects of toxins also depend on the hosting organism. For example, the *Escherichia coli* O157:H7 Shiga toxin (Stx) induces diarrhea, hemorrhagic colitis and hemolytic uremic syndrome in humans, whereas cattle that lack the vascular receptors of Stx are tolerant to the *E. coli* O157:H7 infection [3].

Despite the high frequency of infectious diseases, humans and animals constitute accidental hosts for numerous bacteria and fungi. The two latter are mainly found free or associated to vectors in the environment defining nearby microorganisms as primary targets of bacterial or fungal toxins. During the last decades, several laboratories have focused their interest on environmental reservoir of pathogenic microorganisms. Protozoa, such as FLA, have emerged as important environmental reservoirs of pathogenic bacteria [4,5]. FLA are phagocytic cells widely distributed in the environment that feed on bacteria, fungi, and viruses [6]. Some ingested microorganisms, such as *Legionella pneumophila*, are able to resist amoeba digestion and multiply within FLA [5]. Because of the similarities between amoebae phagocytosis and the digestion by macrophages [7,8], amoeba-resistant microorganisms become resistant to macrophages and potential human/animal pathogens [5]. Worryingly, some bacteria have been shown to become more virulent and more resistant to antimicrobials after their passage through amoebae [9,10]. FLA are also considered to be a melting pot as they allow genetic exchanges between intracellular microorganisms [11]. FLA constitute reservoirs and training grounds of pathogenic microorganisms such as *L. pneumophila* and *Mycobacterium avium* [12,13].

FLA life cycle can exhibit at least two forms: trophozoite and cyst. Being biochemically active, the trophozoite form allows feeding and division. Under adverse environmental conditions, the trophozoite differentiates into a cyst, the resting form [14]. This process, called encystment, is reversible under favorable conditions. In genera such as *Acanthamoeba*, cysts are highly resistant due to a wall containing cellulose and/or chitin [15]. During encystment, bacteria that are trapped within cysts become protected from antimicrobials [16]. For instance, amoeba cysts were theorized to act as survival niches and protective shelters for foodborne pathogenic bacteria such as *E. coli*, *Listeria monocytogenes*, *Salmonella enterica*, and *Yersinia enterocolitica* [17]. Because FLA host, protect, and allow the dissemination of numerous microorganisms in the environment, they could be considered primary targets for toxins. Microorganisms and amoebae interactions could result in environmental selective pressure, thus potentially maintaining or inducing microbial virulence factors and pathogenicity [18]. Virulence factors such as toxins that are selected could then be used against mammalian cells, once the producing microorganisms encounter humans or animals.

Since numerous reviews address interactions between FLA and prokaryotic and eukaryotic microorganisms [5,6,11], this short review will focus on the action of some toxins, effectors or factors, secreted by mammalian pathogens, toward FLA. Molecules of interest that are described here were picked up from well-studied bacteria and fungi. Thus, important parts of the manuscript will be dedicated to the molecules released by the bacterium *L. pneumophila*, which naturally resides in FLA. Effects of toxins and effectors from other airborne pathogens such as the bacterium *Pseudomonas aeruginosa* and the fungus *Aspergillus fumigatus* will be also discussed. The activity of hemolysins produced by foodborne and waterborne pathogens such as *L. monocytogenes* and *V. cholerae* will be addressed. The review will first present the two amoeba models commonly used to study the interaction with bacteria or fungi. Then, the description of microbial secreted molecules that affect amoebae through the inhibition of engulfment and the disturbance of amoeba functions leading to cell death will be elaborated. Finally, the various eukaryotic responses induced by these microbial factors will be addressed.

## 2. Free-Living Amoebae Represent a Relevant Model to Study Bacterial Effectors

At present, studies of crosstalk between bacteria, fungi and environmental amoebae are carried out predominantly with two models from *Dictyostelium* and *Acanthamoeba* genera [19,20,21].

*Acanthamoeba* species are ubiquitously distributed in the environment including soil, water, and air. They are opportunistic pathogens as they are responsible for human diseases such as granulomatous amoebic encephalitis and amoebic keratitis [22]. Most *Acanthamoeba* have two stages during their life cycle: a trophozoite stage, which is the active metabolic form, and a cyst stage, which is the resting and resistant form. The trophozoite presents on its surface spine-like structures named acanthopodia that allow adhesion to surfaces, movement, and prey catching [23]. *Acanthamoeba* feed on microorganisms such as bacteria and fungi [12]. However, some of the organisms ingested resist amoeba digestion. Thus, *Acanthamoeba* spp. represent an important reservoir and vector for various pathogenic bacteria, fungi and viruses [12]. Astonishingly, many pathogens possess the ability to replicate inside *Acanthamoeba* in vitro [13]. Although *Acanthamoeba* spp. are easy to cultivate, genetic manipulations remain challenging. Some genomes of *Acanthamoeba* spp. are available but the polyploidy of this organism is a barrier rendering gene knockouts difficult, even for molecular and cellular biology in general [24,25]. Indeed, only two plasmids seem efficient to transfect *Acanthamoeba castellanii* [26]. Despite these impediments, *Acanthamoeba* spp. remain one of the most commonly used models to study microbial interactions. Numerous publications, that will be presented hereafter, have reported effects of bacterial and fungal products on *Acanthamoeba*.

*Dictyostelium discoideum* is a social amoeba that lives in the soil and feeds on bacteria through phagocytosis. *D. discoideum* has the advantage to be easily grown and genetically tractable with an ease to generate mutants. This amoeba is single-celled but, under starvation, it aggregates with thousands of other solitary cells to become a multicellular moving slug-like organism. This form then produces a fruiting body consisting of a stalk and sorus containing spores. These spores are released and germinate into growing cells thus completing the life cycle [27]. *D. discoideum* interacts with bacteria either at the single-celled or multicellular stage. At the single cell stage, *D. discoideum* adapts its response depending on bacteria in association. Indeed, it was reported as a non-overlapping specificity of the transcriptional response of *D. discoideum* to different bacteria such as *Bacillus subtilis*, *Klebsiella pneumoniae*, *Mycobacterium marinum* and *Micrococcus luteus* [28]. In its aggregative state, to protect the moving slug from toxins or bacterial pathogens, the *D. discoideum* slug has sentinel cells that can engulf bacteria and sequester toxins [29]. These sentinel cells have been shown to clear the bacterium *L. pneumophila* from the slug using a Toll/interleukin-1 receptor (Tir) domain protein, TirA signaling [29]. However, about one-third of the wild *D. discoideum* clones, called farmers, rather than consuming all the available bacteria, keep some and incorporate them into the fruiting body instead. [30]. Arriving in a new area, theses clones are able to seed the stored bacteria as a food source. Farmers have less sentinel cells compared to non-farmers but they also carry non-food bacteria such as *Burkholderia* spp. that renders sentinel cell functionality more efficient [31]. The presence of farmer-associated bacteria prevents farmers from being harmed by toxic molecules, for example, ethidium bromide that is a polycyclic, aromatic compound with a phenanthridine core, which displays toxicity to *D. discoideum* [32]. Interestingly, by mixing farmers and non-farmers of *D. discoideum*, a reduction of spore production in non-farmers has been observed due to chromene, a polycyclic aromatic compound broadly similar to ethidium bromide secreted by *Pseudomonas fluorescens* hosted by non-farmers [33]. Despite the presence of sentinels, the sampling of environmental *D. discoideum* fruiting bodies has revealed important permanent and transient associations with bacteria, underlying a very unique relationship between bacteria and *D. discoideum* far from the simplistic view of predator/prey [34]. As *D. discoideum* is genetically tractable, it offers important potential in elucidating the mode of action of bacterial-secreted toxin on host cells.

Other FLA can be used to study the interaction with other microorganisms. For instance, *Vermamoeba vermiformis* (formerly *Hartmannella vermiformis*) is a ubiquitous thermotolerant amoeba recovered in natural and in man-made water systems. The growing interest in this amoeba lies in the fact that *V. vermiformis* is highly abundant in hospital water systems. Similarly to *Acanthamoeba*, *V. vermiformis* present a two-stage life and were also shown to host numerous microorganisms including bacteria and fungi [35].

FLA of the *Naegleria* genus are also used as a model to study interactions between FLA and microorganisms. *Naegleria* spp. are commonly found in soils and freshwater. This genus is composed of more than 40 species among which the amoeboflagellate *Naegleria fowleri*, responsible for the human primary amoebic encephalitis, can be found [36]. Most of *Naegleria* exist in three distinct stages: a trophozoite active form, a swimming flagellate state, and a resting cyst form. This model is highly used in association with the bacterium *L. pneumophila* [37].

## 3. Bacteria and Fungi Can Secrete Surface Molecules That Prevent Amoeba Engulfment

In the environment, bacterial communities are regulated by FLA [6]. To prevent physical interactions, certain microorganisms produce anti-amoeba molecules on their membrane. Thus, the surface exposure of the green pigment 1,8-dihydroxynaphthalene-melanin protects the fungi *A. fumigatus* from phagocytic uptake and intracellular killing by the amoeba, *Protostelium aurantium,* and delays its exocytosis from *D. discoideum* [38]. Similar effects were observed with the bacterium *V. cholerae* for which production of the pyomelanin pigment allows resistance to predation by *A. castellanii* [39]. The protective role for pigments comes as a supplement to the bacterial surface structures such as lipopolysaccharide (LPS) and outer membrane proteins. In the case of *K. pneumoniae*, the LPS and the outer membrane proteins, OmpA and OmpK36, seem critical to counteract predation by *D. discoideum* [40]. If PagP-dependent lipid A palmitoylation contributes to reducing *Klebsiella* engulfment by *D. discoideum*, the homologous gene (pagP-like) in *L. pneumophila* promotes the intracellular infection in the amoeba *V. vermiformis* [41]. *A. castellanii* was reported to bind the *E. coli* LPS carbohydrate using mannose-binding protein located on their surface. Recognition of *E. coli* by *A. castellanii* seems inhibited by O1 and not O157 O-antigen types [42]. Interestingly, the mannose binding protein sequence from *A. castellanii* has no similarity with other metazoan mannose binding proteins [42]. Chlamydiae represent a group of bacteria containing human pathogenic organisms and several endosymbionts in amoebae. Chlamydiae such as the abortigenic *Waddlia chondrophila*, can resist predation by *A. castellanii* using effectors secreted by the Type 3 Secretion System such as Wimp1 (Wcw_1131). This putative Ras guanine-nucleotide exchange factor, expressed early during the course of a replication cycle, localizes to the inclusion membrane of *W. chondrophila* [43]. These Chlamydial Inclusion membrane proteins (Incs) are suggested to induce the formation of the inclusion membrane, important for the chlamydial developmental cycle [44]. The presence of Incs was also reported during the multiplication of the endosymbiont *Protochlamydia amoebophila* in *A. castellanii* [45].

## 4. Pathogenic Bacteria Use Toxins or Secreted Factors to Disturb Amoeba Functions to Survive

Bacteria have evolved various anti-predator defense strategies to resist and survive [46]. The bacterium *L. pneumophila* uses the *Legionella* collagen-like protein (Lcl) to induce an autoaggregation that favors *Legionella* attachment and invasion of *A. castellanii* and *V. vermiformis* [47]. The activity of the putative adenylate cyclase LadC is important in the ability of *L. pneumophila* to infect *A. castellanii* [48]. The aminopeptidase LapA and the acyltransferase PlaC, two effectors from the type II secretion system (T2SS), are involved in nutrient acquisition during *L. pneumophila* infection in *A. castellanii* [49]. *Legionella* also uses the type IV secretion system effector Ankyrin B (AnkB) to generate nutrients. AnkB is a F-box-containing protein that interacts with the host SCF1 ubiquitin ligase complex. It is the only known Dot/Icm-translocated nutritional virulence effector of *L. pneumophila* essential for acquisition of Lys48-linked polyubiquitinated proteins by the *Legionella*-containing vacuole (LCV) within *D. discoideum* and *Acanthamoeba polyphaga* [50]. Anchorage of AnkB to the cytosolic face of LCV is mediated by the host farnesylation machinery. The proteasomal degradation of AnkB-assembled polyubiquitinated proteins generates amino acids essential for the robust intra-vacuolar proliferation of *L. pneumophila* [51]. *Legionella* interferes with ubiquitination through multiple processes. The *Legionella* effectors belonging to the SidE family are required for virulence of *L. pneumophila* within *A. castellanii* and *D. discoideum* by catalyzing the phosphoribosyl-linked serine ubiquitination of Rab33b [52]. SidJ was shown to be essential for *Legionella* growth in *D. discoideum* [53,54]. If SidE proteins function as toxins during early stages of infection, it has been shown that the effector SidJ inactivates them by mediating their removal from the surface of *Legionella*-containing vacuoles [54]. SidJ is a calmodulin-dependent glutamylase that mediates both glutamylation of several host proteins and antagonizes the Ubiquitin ligase activity of the *Legionella* effector SdeA by modifying the SdeA catalytic glutamate in the mono-ADP ribosyl transferase domain [55]. Other effectors from *Legionella* contribute to the bacterial replication within amoebae. The Sel1 Repeat Protein LpnE is required for *L. pneumophila* to infect *A. castellanii* [56]. LpnE might bind or stabilize the Oculocerebrorenal syndrome of Lowe (OCRL1) on *Legionella*-containing vacuoles (LCV) to promote the restriction of the intracellular growth of *L. pneumophila*. It was suggested that the interaction between LpnE and OCRL1 could represent a bacterial mechanism to downregulate intracellular replication in order to sustain the protective niche and to avoid rapid killing of the host cell [57]. Indeed, OCRL1 is an Inositol polyphosphate 5-phosphatase that regulates retrograde vesicle trafficking between endosomes and the Trans-Golgi network. The effector ProA likely acts after the onset of replication to promote intracellular replication of *Legionella* [58]. This effector is translocated out of the LCV into the host cytoplasm where it likely acts on a wide range of targets [59], as well as cleaving and activating other T2SS-dependent exoenzymes [49]. By cleaving and activating effectors such as LapA and PlaC, ProA contributes to the acquisition of amino acids by *L. pneumophila*. In order to obtain nucleotides and phosphate, the T2SS effector SrnA that exhibits ribonuclease activity is involved in the degradation of RNA from *V. vermiformis* and *Naegleria lovaniensis* [60]. Effectors LepA and LepB were shown to allow nonlytic release of *L. pneumophila* from *A. castellanii* [61].

Similarly to *Legionella*, *Salmonella* and *Pseudomonas* express toxins with ADP-ribosylation activities. Regarding *Salmonella*, the pathogenicity island (SPI) 1 and 2-encoded type III secretion systems are essential for the survival of *S. enterica* within *Acanthamoeba rhysodes* and *A. polyphaga* [62,63]. *A. rhysodes* infected with *S. enterica* displayed a modification of morphology and a loss of adherence. These effects could result from the ADP-ribosylation of actin by the *Salmonella* enzyme SpvB [64]. As found with the *Clostridium perfringens* Iota toxin, ADP-ribosylation by SpvB activates actin degradation in *A. rhysodes* [64]. *P. aeruginosa* is protected against protozoan grazing through quorum sensing mediated gene expression by promoting the formation of microcolonies and production of alginate [65]. *Pseudomonas* has been shown to inject several type III effectors into eukaryotes, among which ExoU, a phospholipase A2 activator [66]; ExoS and ExoT enzymes that can disrupt the actin cytoskeleton and inhibit host autophagy through their N-terminal small GTPase-activating protein (GAP) domain and C-terminal ADP-ribosyltransferase (ADPRT) domain [67].

The bacterium *Yersinia* also translocates a *Yersinia* outer protein (Yop), with GTPase activating protein (GAP) activity, into the host cell via a type III secretion system. YopE is a GAP for RhoA, Rac1, Cdc42 and RhoG [68,69]. In *D. discoideum*, the ectopic expression of YopE impairs Rac1 and possibly also RacH activation suggesting that GTPases might be affected by YopE in very wide models [70].

The bacterium *M. marinum* secretes tyrosine phosphatases PtpA, PtpB, and the secretory acid phosphatase SapM that promotes formation of the *Mycobacterium*-containing vacuole (MCV) and then the vacuolar escape in *D. discoideum* and *A. castellanii* [71]. These phosphatases hydrolyse the phosphatidylinositol 3-monophosphate and the PtpA prevents acidification of MCV by restricting the accumulation of the V-ATPase proton pump on MCV.

## 5. The Host Cell Death Is a Common Read-Out Induced by Toxins

Although *E. coli* O157 can be recognized by *Acanthamoeba*, it was reported to survive and replicate in *A. polyphaga* [72]. *E. coli* O157:H7 expressing the Shiga toxin Stx was shown to induce *A. castellanii* cell death [73,74]. It was reported that *E. coli* producing the Shiga toxin that were isolated from infected cows compared to strains isolated from humans were more efficient at killing *A. castellanii,* highlighting the importance of the Stx isoforms [73]. A study shows that the carriage of the Stx-encoding prophage increases the survival of *E. coli* in the food vacuoles of protozoa such as *Tetrahymena pyriformis,* whereas other publications did not find any protective effect of Stx [75,76]. The role of Stx in protozoan predation requires further investigation.

The *P. aeruginosa* effectors ExoS, ExoT and ExoU contribute to *A. castellanii* killing [77,78]. Cytotoxicity of ExoU was also observed in *D. discoideum* [79]. Rhamnolipids secreted by *P. aeruginosa* also contributes to the fast lysis of *D. discoideum* [80].

In the case of *L. monocytogenes*, this bacterium was reported to be ingested by and survive within protozoa such as *Acanthamoeba* spp. or *Tetrahymena pyriformis* [81]. The expression of pore-forming toxin Listeriolysin O (LLO) by *Listeria* was shown to cause a decrease in the *Amoeba proteus* population [82]. Similarly to mammalian cells, LLO seemed to induce perforation of the phagosomal membrane in *A. proteus* [82,83]. Although LLO was reported to favor *Listeria* growth and encystment of the ciliate *T. pyriformis* [82], these effects could be dependent on the experimental conditions. Indeed, bacteria could grow saprophytically on materials released from amoebae extracellularly, [84] and some studies reported that *L. monocytogenes* does not actively kill *A. castellanii* with weak evidence that LLO can contribute to improved *Listeria* survival after ingestion by *A. castellanii* [85]. *L. monocytogenes* seem unable to survive within *A. polyphaga* and *A. lenticulata* irrespective of the presence of LLO (*hly*) coding gene [86,87,88].

Infection of *A. castellanii* by *M. marinum* results in lysis of the amoeba host in an exporter ESX-1-dependent manner [89]. This virulence is associated with the export of EsxA (ESAT-6) and EsxB (CFP-10) to the surface of the bacterium [90]. In both *M. marinum* and *M. tuberculosis*, EsxA exhibits acidic pH-dependent membrane-permeabilizing activity contributing to the mycobacterial intracellular survival [91].

Once stressed, FLA differentiate to their cyst form, which is highly resistant to various adverse conditions. The bacterium *V. cholerae* was shown to use a lecithinase enzyme to permeabilize *A. castellanii* plasma membrane thus inducing lysis of cysts [92].

Similarly to bacteria, fungi are also able to induce amoeba lysis. The pathogenic fungus *A. fumigatus* is able to induce *D. discoideium* lysis through secretion of the non-ribosomal immunosuppressive epipolythiodioxopiperazine gliotoxin [93]. The latter was shown, in human polymorphonuclear leukocytes, to abrogate p47phox phosphorylation that is important in assembling an active NADPH oxidase complex [94]. *Aspergillus* was also found to produce fumagillin (H-3), a toxin with antiphage activity that has amoebicidal properties toward *N. fowleri* and *Entamoeba histolytica* [95,96].

## 6. Toxins and Microbial Secreted Factors Induce Various Responses That Depends on the Host

Toxins modulate host-specific processes. For example, Diphteria toxin (DT) is a protein from *Corynebacterium diphtheriae* that was shown to cross the endosomal membrane, to catalyze the NAD+-dependent ADP-ribosylation of elongation factor 2 and to inhibit protein synthesis [97]. Although the DT causes a cytotoxic effect on mammalian cells, it did not inhibit *Acanthamoeba*. The lack of anti-amoeba toxicity could be attributed to a weak binding to the *Acanthamoeba* membrane or to a defective transport mechanism [98].

In the case of *Legionella*, this bacterium modulates mammalian-specific processes that are absent from amoebae, such as programmed cell death, or phosphorylation and activation of NF- κB by LnaB and LegK1 in mammalian hosts [99,100,101,102,103]. The action of the *Legionella* SidE effector could also be host specific. Indeed, Sde proteins target host reticulon 4 (Rtn4) to control tubular ER dynamics [104]. Although Rtn4 and Rab33b homologs can be found in the available genomes of *D. discoideum*, *Tetrahymena thermophila*, and *Naegleria gruberi*, they seem absent in other *Tetrahymena* spp., *Naegleria* spp., and *Vermamoeba* spp. [105]. Several other effectors were reported to be specific to a wide-range of hosts. PlaC is a glycerophospholipid:cholesterol acyltransferase that has phospholipase A and lysophospholipase activities [106]. It was suggested that PlaC alters ergosterol-containing membranes of *V. vermiformis* and *N. lovaniensis* but not *A. castellanii* [107]. It is possible that the fatty acid profile and position of fatty acids in glycerophospholipids may be different in the different amoebae. The role of ProA effector in *V. vermiformis* infection, not with *N. lovaniensis*, could be to activate PlaC. The effector NttA promotes *Legionella* growth in *A. castellanii* and *Willaertia magna* but not in *V. vermiformis*, *N. lovaniensis* or macrophages [58]. SrnA and PlaC effectors were shown to be necessary for optimal intracellular multiplication in *V. vermiformis* but not *A. castellanii* [108]. SrnA was not essential for bacteria growth in macrophages [60]. NttC was required for infection of *V. vermiformis* and *W. magna* but not for infection of *N. lovaniensis* or *A. castellanii* [109]. The effector NttD was required for infection of *A. castellanii* but not in human macrophages, *Naegleria* and *V. vermiformis* [49]. A recent study has shown that *L. pneumophila* is able to inject an amylase (LamA), into the cytosol of both human macrophages and *A. polyphaga* to both degrade glycogen and generate cytosolic hyper-glucose. LamA-mediated glycogenolysis deprives *Acanthamoeba* of the main building blocks for the synthesis of the carbohydrates-rich cyst wall. This activity prevents the encystment of *A. polyphaga* and promotes a permissive host. Interestingly, LamA induces a shift in macrophage metabolism to aerobic glycolysis, which triggers an M1-like pro-inflammatory differentiation that restricts *Legionella* proliferation ex and in vivo [110]. Regarding host cell death, it seems that *L. pneumophila* genes related to pyroptosis (flaA and sdhA) and apoptosis (vipD and sidF) are differently regulated between THP1 monocytes and *A. castellanii* [111]. Authors suggested that *Legionella* is better adapted to *A. castellanii* than to macrophages and it more readily induces cell death in *A. castellanii* compared to monocytes.

The *P. aeruginosa* toxin L-2-amino4-methoxy-trans-3-butenoic acid inhibits the growth of *A. castellanii*. The putative stress induced by this toxin activates encystment in *A. castellanii*. This effect could depend on the context or the presence of other virulence factors as the L-2-amino4-methoxy-trans-3-butenoic acid production by *P. aeruginosa* does not alter grazing resistance toward *A. castellanii* [112].

Using the secreted hemolysin HlyA, *V. cholerae* disturbs *A. castellanii* functions with aberrant amoeba morphology and impairment of encystment. This effect was shown to be counteracted by the *Vibrio* HapA protease which probably cleaves HlyA to protect the host *Vibrio*-colonized Contractile Vacuole from premature lysis. Interestingly, in the same conditions, neither the RTX and VasX pore-forming toxins nor the T6SS contribute to *V. cholerae*’s interaction with *A. castellanii* [92]. Differing from *A. castellanii*, the T6SS is involved in the cytotoxicity of *V. cholerae* toward *D. discoideum* and macrophages [113]. *V. cholerae* requires the putative lipase TseL and the phospholipid-interacting protein VasX to kill *D. discoideum* by a mechanism that depends on actin cross-linking [114,115,116].

*M. marinum* produces siderophores such as a lipid-bound mycobactin (MBT) and a water-soluble variant carboxymycobactin (cMBT). Purified cMBT promotes the bacterial growth in macrophages but not in *A. castellanii* [117]. *Mycobacterium abscessus* expresses a Phospholipase C within *A. castellanii* [118]. This expression was necessary for the intra-amoebal growth of *M. abscessus*. While PLC induces membrane phospholipid degradation and DAG production, its activity did not confer a supplementary advantage to *M. abscessus* within macrophages [118].

The non-germinating spores of *A. fumigatus* can also produce diffusates which inhibit the growth of *N. gruberi*, an effect that was not observed on *A. castellanii* [119].

Beyond toxins, the set of bacterial genes involved in interactions with amoebae or macrophages could differ. Hence, in contrast to MDCK epithelial cells, the key regulator in the expression of the invasion-associated SPI-1, hilA, is dispensable for *Salmonella* uptake by *A. rhysodes* [63]. Another example, the SPI-1 gene, *sipC,* is induced during *A. polyphaga* infection while it is downregulated in murine macrophage-like cells [62]. Regarding *Bordetella*, the master virulence regulatory system BvgAS senses environmental cues and controls the expression of virulence factors [120]. Genes expressed in the Bvg positive (Bvg+) phase are essential for the infection of the mammalian respiratory tract [121], while those associated with the Bvg-phase are important for *B. bronchiseptica* growth and dissemination via the fruiting body sori of *D. discoideum* [122]. *Yersinia pestis* can be phagocytized by *A. castellanii* trophozoites. However, induction of the T3SS was shown to prevent the phagocytic uptake of *Y. pestis* by *A. castellanii* [123]. Thus, in the environment, the absence of T3SS induction due to low temperature would serve to enhance phagocytosis of *Y. pestis*, while, in mammalian cells, the T3SS induced by a temperature of 37 °C allows inhibition of macrophage phagocytosis [124]. Amoebae, *A. castellanii* and *V. vermiformis,* favor the growth of spores from the *Bacillus anthracis*. Unlike macrophages, germination and growth of *B. anthracis* does not depend on the germination receptor *gerX* gene [125,126].

## 7. Conclusions

Amoebae can be considered primary targets of bacterial and fungal toxins. In the environment, amoeba phagocytosis imposes major selection pressure on bacteria and fungi toward the acquisition of virulence factors that are used to escape phagocytosis or elimination by mammalian phagocytes. Phenotypes or activities induced by toxins in amoebae cannot always be translated to mammalian host cells. There are differences between mammalian cells and protozoan responses to bacteria and toxins which depend on a multitude of factors such as host surface receptors and various intracellular machineries. Regarding mammalian pathogens, the various responses induced by the toxins they express should push us to extend our investigations beyond mammalian cells toward their natural predators, which are free-living amoebae. Indeed, many questions such as “how do the toxins subverting the host immune response affect FLA?” or “through which mechanisms can bacteria or fungi produce distinct toxins or effectors depending their host?” remain under-investigated.

## Data Availability

Data sharing not applicable.
